# Association Between Plasma Caffeine and Other Methylxanthines and Metabolic Parameters in a Psychiatric Population Treated With Psychotropic Drugs Inducing Metabolic Disturbances

**DOI:** 10.3389/fpsyt.2018.00573

**Published:** 2018-11-09

**Authors:** Aurélie Delacrétaz, Frederik Vandenberghe, Anaïs Glatard, Axel Levier, Céline Dubath, Nicolas Ansermot, Séverine Crettol, Mehdi Gholam-Rezaee, Idris Guessous, Murielle Bochud, Armin von Gunten, Philippe Conus, Chin B. Eap

**Affiliations:** ^1^Unit of Pharmacogenetics and Clinical Psychopharmacology, Centre for Psychiatric Neuroscience, Department of Psychiatry, Lausanne University Hospital, Prilly, University of Lausanne, Lausanne, Switzerland; ^2^Centre of Psychiatric Epidemiology and Psychopathology, Department of Psychiatry, Lausanne University Hospital, Prilly, University of Lausanne, Lausanne, Switzerland; ^3^Unit of Population Epidemiology, Department of Community Medicine and Primary Care and Emergency Medicine, University Hospital of Geneva, Geneva, Switzerland; ^4^Division of Chronic Diseases, Institute of Social and Preventive Medicine, Lausanne University Hospital, University of Lausanne, Lausanne, Switzerland; ^5^Service of Old Age Psychiatry, Department of Psychiatry, Lausanne University Hospital, Prilly, University of Lausanne, Lausanne, Switzerland; ^6^Service of General Psychiatry, Department of Psychiatry, Lausanne University Hospital, Prilly, University of Lausanne, Lausanne, Switzerland; ^7^School of Pharmaceutical Sciences, University of Geneva, Geneva, Switzerland

**Keywords:** psychotropic drugs, lipids, plasma caffeine level, plasma methylxanthines, metabolic parameters, secondary effects

## Abstract

**Importance:** Multiple studies conducted in the general population identified an association between self-reported coffee consumption and plasma lipid levels. To date, no study assessed whether and which plasma methylxanthines (caffeine and/or its metabolites, i.e., paraxanthine, theophylline, and theobromine) are associated with plasma lipids. In psychiatric patients, an important coffee consumption is often reported and many psychotropic drugs can induce a rapid and substantial increase of plasma lipid levels.

**Objective:** To determine whether plasma methylxanthines are associated with metabolic parameters in psychiatric patients receiving treatments known to induce metabolic disturbances.

**Design, Setting, and Participants:** Data were obtained from a prospective study including 630 patients with metabolic parameters [i.e., body mass index (BMI), total cholesterol (TC), low-density lipoprotein cholesterol (LDL-C), high-density lipoprotein cholesterol (HDL-C), non-high-density lipoprotein cholesterol (non-HDL-C), and fasting triglycerides (TG)] monitored routinely during psychotropic treatment.

**Exposures:** Plasma methylxanthines levels.

**Main Outcomes and Measures:** Metabolic variables including BMI and plasma lipid levels.

**Results:** Multivariate analyses indicated that BMI, TC, HDL-C, and non-HDL-C increased significantly with increasing total methylxanthines (*p*_corrected_ ≤ 0.05). In addition, compared to patients with plasma caffeine concentration in the lowest quartile, those with caffeine concentration in the highest quartile were twice more prone to suffer from non-HDL hypercholesterolemia (*p*_corrected_ = 0.05), five times more likely to suffer from hypertriglyceridemia (*p*_corrected_ = 0.01) and four times more susceptible to be overweight (*p*_corrected_ = 0.01).

**Conclusions and Relevance:** This study showed that plasma caffeine and other methylxanthines are associated with worsening of metabolic parameters in patients receiving psychotropic treatments known to induce metabolic disturbances. It emphasizes that important caffeine consumption could be considered as an additional environmental risk factor for metabolic worsening in patients receiving such treatments.

## Introduction

Coffee is one of the most commonly consumed beverages worldwide and its influence on multiple human health features has been extensively demonstrated (1). A large body of epidemiological studies has examined the association of coffee consumption with various diseases, often yielding inconsistent results. Recent reviews integrating numerous meta-analyses have drawn comprehensive conclusions on the overall effects of coffee consumption. In particular, one recent umbrella review, i.e., a review gathering existing research syntheses of different outcomes for the same intervention, showed that coffee consumption has beneficial effects on a broad range of chronic diseases including cancers, neurological diseases, and cardiovascular diseases (2). However, potential detrimental effects of caffeine consumption on certain health outcomes were also reported, as for instance on serum lipids.

For the last 30 years, multiple epidemiological studies considering association between coffee consumption and serum lipids resulted in inconsistent findings (3–18). More recently, a meta-analysis identified significant positive associations between coffee consumption and serum lipid levels in population-based cohorts, despite an important heterogeneity among considered studies (19). Thus, on average, drinking coffee for 45 days was associated with significant increases of total cholesterol (TC), low-density lipoprotein cholesterol (LDL-C), and triglyceride (TG) of 8.1 mg/dl (0.21 mmol/l), 5.4 mg/dl (0.14 mmol/l), and 12.6 mg/dl (0.14 mmol/l), respectively (19). Interestingly, a dose-response influence of coffee intake on serum lipid levels was recognized, suggesting that the influence of caffeine and/or other coffee components on blood lipid levels may be dose-dependent. Consistent with this hypothesis, methylxanthines including caffeine and its metabolites (i.e., paraxanthine, theobromine, and theophylline) were related with multiple biological effects such as an increased fat oxidation, mobilization of glycogen in muscle as well as an increased lipolysis (20, 21). However, methylxanthines are found in many other widespread-consumed products than coffee (e.g., tea, energy drinks, and chocolate) (22) and their concentration in coffee is highly variable (23). Thus, as previous studies all considered self-reported coffee consumption to evaluate the influence of caffeine and of other methylxanthines on various outcomes of interest, the observed effects may not have been sufficiently accurate.

In psychiatric patients, an important coffee consumption has been reported (24), and higher rates of high caffeine consumers were observed in psychiatric patients compared to individuals from the general population (25–27), which may enhance the risk of developing hyperlipidemia. Sedative and/or anticholinergic effects induced by psychotropic drugs may constitute one possible argument to explain a higher coffee consumption in psychiatric patients than in the general population. In addition, the use of psychotropic medications such as antipsychotics (most atypical but also some typical), mood stabilizers (e.g., lithium and valproate), and some antidepressants (e.g., mirtazapine) can increase the risk of developing metabolic disorders including obesity and dyslipidemia (28–31). These factors, combined with additional risk factors including psychiatric disease-related factors, unhealthy lifestyle, and poverty (32, 33), may therefore contribute to the excessive susceptibility of psychiatric patients for developing cardiovascular diseases.

To the best of our knowledge, no study has yet investigated whether plasma methylxanthine levels are associated with plasma lipid levels and body mass index (BMI), neither in the general nor in the psychiatric population. In the present study, we measured plasma levels of caffeine and its metabolites in a large cohort of psychiatric patients receiving psychotropic treatments known to induce metabolic disturbances, aiming to examine whether plasma methylxanthine levels are associated with lipid parameters and BMI.

## Methods

### Study design

Since 2007, a longitudinal observational study (PsyMetab) has been ongoing at the Department of Psychiatry of the Lausanne University Hospital as described elsewhere (34). Patients with informed consent starting or already receiving a psychotropic treatment known to have a potential risk to induce metabolic disturbances (i.e., antipsychotics, mood stabilizers, and some antidepressants, as listed in S1 Table) were included, as described in the flowchart (S1 Figure). More information in Supplementary Material.

### Lipid and methylxanthine parameters

The majority of blood samples (i.e., 66%) were drawn in fasting conditions. Non-fasting blood samples (i.e., within 6 h following last meal) were excluded only for triglyceride analysis (not for total, HDL- and LDL-cholesterol) (35, 36). LDL-cholesterol was calculated using the Friedewald formula only if triglyceride levels were lower than 3.5 mmol/l (37, 38). In the present study, dyslipidemia was defined as hypercholesterolemia (TC ≥ 5 mmol/l) and/or HDL hypocholesterolemia (HDL-C < 1 mmol/l) and/or LDL hypercholesterolemia (LDL-C ≥ 3 mmol/l) and/or hypertriglyceridemia (TG ≥ 2 mmol/l) and/or if patients were treated with lipid-lowering drugs (S2 Table). More information on quantification of lipids and of methylxanthines (i.e., caffeine, paraxanthine, theophylline, and theobromine) is available in Supplementary Material.

### Statistical analyses

Wilcoxon-Mann-Whitney rank-sum tests and Chi-squared tests were conducted to compare continuous and categorical variables, respectively, across patient groups (e.g., patients stratified by methylxanthine quartiles). Plasma levels of methylxanthine were log-transformed to reduce their variability. The xtmixed procedure in Stata 14 was used to fit linear mixed effect models on plasma lipid levels adjusting for age, sex, smoking status, psychotropic medication, treatment duration, BMI (whenever appropriate), and plasma methylxanthine levels. The fitted linear mixed effect model had a random effect at the subject level.

In order to determine whether and which stratified analyses could be conducted, statistical interactions of possible confounding factors [i.e., age, gender, smoking status, psychotropic medication, treatment duration, and BMI (or TC whenever applicable)] with methylxanthine levels on metabolic outcomes (i.e., lipid levels and BMI) were tested by including interaction terms in linear mixed models adjusting for covariates.

Odds ratio of disturbed metabolic outcomes (e.g., dyslipidemia and overweight) by quartiles of plasma methylxanthines were conducted using logistic mixed effects models adjusting for age, sex, smoking status, psychotropic medication, treatment duration, and BMI or TC, whenever applicable.

Statistical significance was defined as a *p*-value ≤ 0.05. *P*-values were adjusted for multiple testing using the false discovery rate (FDR) approach. Statistical analyses were performed using Stata 14 (StataCorp, College Station TX, USA) and R environment for statistical computing version 3.3.1.

## Results

### Demographics and plasma levels of lipids and methylxanthines

Demographic and clinical characteristics of the psychiatric sample are displayed in Table 1. Six hundred and thirty patients without missing data were included. Median age was 39 years [interquartile range (IQR): 27–54], psychotic disorders (F20–F24; F28; F29) were the most frequent diagnosis (49%) and quetiapine was the most frequently prescribed psychotropic drug (29%). Half of the patients smoked (50%) and 70% of the selected observation for each single patient was collected within the first 6 months of treatment.

**Table 1:** Demographic parameters and plasma levels of lipids and of methylxanthines in patients receiving psychotropic drugs.

	**Total *n* = 630**
Male, *n* (%)	327 (51.9)
Age, median (IQR) (years)	39 (27–54)
Main diagnosis, *n* (%)	
Organic mental disorders (F0)	14 (3.0)
Psychotic disorders (F20–F24, F28, F29)	232 (48.8)
Schizoaffective disorders (F25)	48 (10.1)
Bipolar disorders (F30–F31)	83 (17.5)
Depressive disorders (F32–F33)	98 (20.6)
Psychotropic medication, *n* (%)
Amisulpride	60 (9.5)
Aripiprazole	61 (9.7)
Asenapine	1 (0.2)
Clozapine	32 (5.1)
Haloperidol	2 (0.3)
Lithium	36 (5.7)
Lurasidone	1 (0.2)
Mirtazapine	21 (3.3)
Olanzapine	92 (14.6)
Quetiapine	181 (28.7)
Risperidone	130 (20.6)
Valproate	12 (1.9)
Zuclopenthixol	1 (0.2)
Smoking, *n* (%)
Yes	314 (49.8)
No	316 (50.2)
Duration of psychotropic treatment, *n* (%) (months)
Baseline	62 (9.8)
1	227 (36.0)
2–5	152 (24.1)
≥6	189 (30.0)
Total cholesterol, median (IQR) (mmol/l) (*n* = 630)	5.0 (4.3–5.7)
LDL-C, median (IQR) (mmol/l) (*n* = 602)	2.9 (2.3–3.6)
HDL-C, median (IQR) (mmol/l) (*n* = 623)	1.4 (1.1–1.6)
Fasting TG, median (IQR) (mmol/l) (*n* = 424)	1.2 (0.9–1.8)
Non-HDL-C, median (IQR) (mmol/l) (*n* = 623)	3.6 (2.9–4.4)
BMI, median (IQR) (kg/m^2^) (*n* = 630)	24.2 (21.5–28.4)
Caffeine, median (IQR) (ng/ml)	416 (84–1,513)
Paraxanthine, median (IQR) (ng/ml)	507 (164–1,178)
Theophylline, median (IQR) (ng/ml)	75 (31–174)
Theobromine, median (IQR) (ng/ml)	626 (256–1,301)
Total methylxanthines, median (IQR) (ng/ml)	2,002 (805–4,195)

*Patients with available age, sex, smoking status, psychotropic medication, treatment duration, BMI and plasma levels of lipids and of methylxanthines, and who did not receive any lipid lowering treatment were included in analyses. Only the first observation per patient was included. IQR, interquartile range; LDL-C, low-density lipoprotein cholesterol; HDL-C, high-density lipoprotein cholesterol; TG, triglyceride; non-HDL-C, non-high-density lipoprotein cholesterol; BMI, body mass index*.

The evolution of metabolic parameters and of methylxanthine plasma levels during treatment with psychotropic drugs is presented in S3 Table and in Table 2, respectively. Although patients might differ across periods of treatment, a significant worsening of TC, LDL-C, and non-HDL-C levels occurred during treatment with psychotropic drugs. Thus, TC, LDL-C levels, and non-HDL-C increased from 4.8, 2.8, and 3.4 mmol/l at baseline to 5.1, 3.0, and 3.7 mmol/l after 6 months of treatment (*p* = 0.02, 0.03, and 0.01), respectively. Of note, no significant worsening was observed for HDL-C or TG, possibly attributable to an insufficient power due to the lower number of observations and/or to a lower variation of these parameters. Interestingly, plasma levels of total methylxanthines increased significantly during treatment with psychotropic drugs (*p*_corrected_ ≤ 0.05).

**Table 2:** Evolution of metabolic (including lipid) and of methylxanthine parameters during psychotropic treatment known to induce metabolic disturbances.

	***n***	**Baseline**	***n***	**1 month**	***p*${}_{\text{corrected}}^{\text{a}}$**	***n***	**2–5 months**	***p*${}_{\text{corrected}}^{\text{b}}$**	***n***	**≥ 6 months**	***p*${}_{\text{corrected}}^{\text{c}}$**
Caffeine, median (IQR) (ng/ml)	129	275 (68–1,133)	343	343 (93–1,301)	0.29	402	455 (96–1,463)	0.08	400	476 (111–1,656)	0.06
Paraxanthine, median (IQR) (ng/ml)	129	335 (118–931)	343	448 (150–1,046)	0.25	402	515 (144–1,167)	0.08	400	506 (186–1,128)	0.06
Theophylline, median (IQR) (ng/ml)	129	130 (57–725)	343	72 (31–165)	0.2	402	83 (29–168)	0.07	400	78 (33–168)	0.06
Theobromine, median (IQR) (ng/ml)	129	417 (161–965)	343	577 (256–1,350)	**0.005**	402	664 (308–1,194)	**0.002**	400	607 (257–1,197)	**0.01**
Total methylxanthines, median (IQR) (ng/ml)	129	1,338 (512–3,351)	343	2003 (756–4,128)	**0.05**	402	2,009 (871–4,128)	**0.01**	400	1,963 (853–4,208)	**0.01**

*Patients with available age, sex, smoking status, psychotropic medication, treatment duration, BMI and plasma levels of lipids and of methylxanthines, and who did not receive any lipid lowering treatment were included in the analyses. If multiple follow-ups per patient were available, only data from the first follow-up was considered*.

*^a^p-values were calculated using ranksum tests (for continuous variables) and chi2 tests (for categorical variables) between baseline vs. 1 month of treatment. FDR correction was applied. Values in bold are significant*.

*^b^p-values were calculated using ranksum tests (for continuous variables) and chi2 tests (for categorical variables) between baseline vs. from 2 to 5 months of treatment. FDR correction was applied. Values in bold are significant*.

*^c^p-values were calculated using ranksum tests (for continuous variables) and chi2 tests (for categorical variables) between baseline vs. ≥6 months of treatment. FDR correction was applied. Values in bold are significant*.

Methylxanthines increased significantly with increasing age (*p*_corrected_ ≤ 0.005, S4 Table and S2 Figure) except theobromine. S4 Table shows that women were more prone to have high levels of all methylxanthines but theobromine (*p*_corrected_ ≤ 0.04). More interestingly, BMI and all plasma lipid levels but LDL-C significantly increased with increasing quartiles of all methylxanthines (*p*_corrected_ ≤ 0.05), except for theobromine. S3.1–S3.6 Figures display distribution of plasma lipid levels and of BMI according to plasma levels of methylxanthines. In accordance with results from methylxanthine quartiles analyses, BMI, and plasma levels of TC, LDL-C, TG, and non-HDL-C increased with increasing levels of methylxanthines, while the association between HDL-C and methylxanthine levels was less apparent. Of note, prevalence of hypercholesterolemia and overweight increased significantly with increasing quartiles of all methylxanthines but theobromine (*p*_corrected_ ≤ 0.04; S5 Table). S6 Table shows that neither diagnosis nor psychotropic medication groups were associated with methylxanthine levels. Finally, trends of higher levels of methylxanthines except theobromine were observed in patients receiving clozapine or olanzapine [i.e., drugs with high concomitant affinities for AchM and H1 receptors (39)] as compared to patients who received drugs with less potent anticholinergic and sedative effects (*p*_corrected_ = 0.06, S7 Table).

### Associations of plasma methylxanthines with metabolic parameters

**Figure 1:**
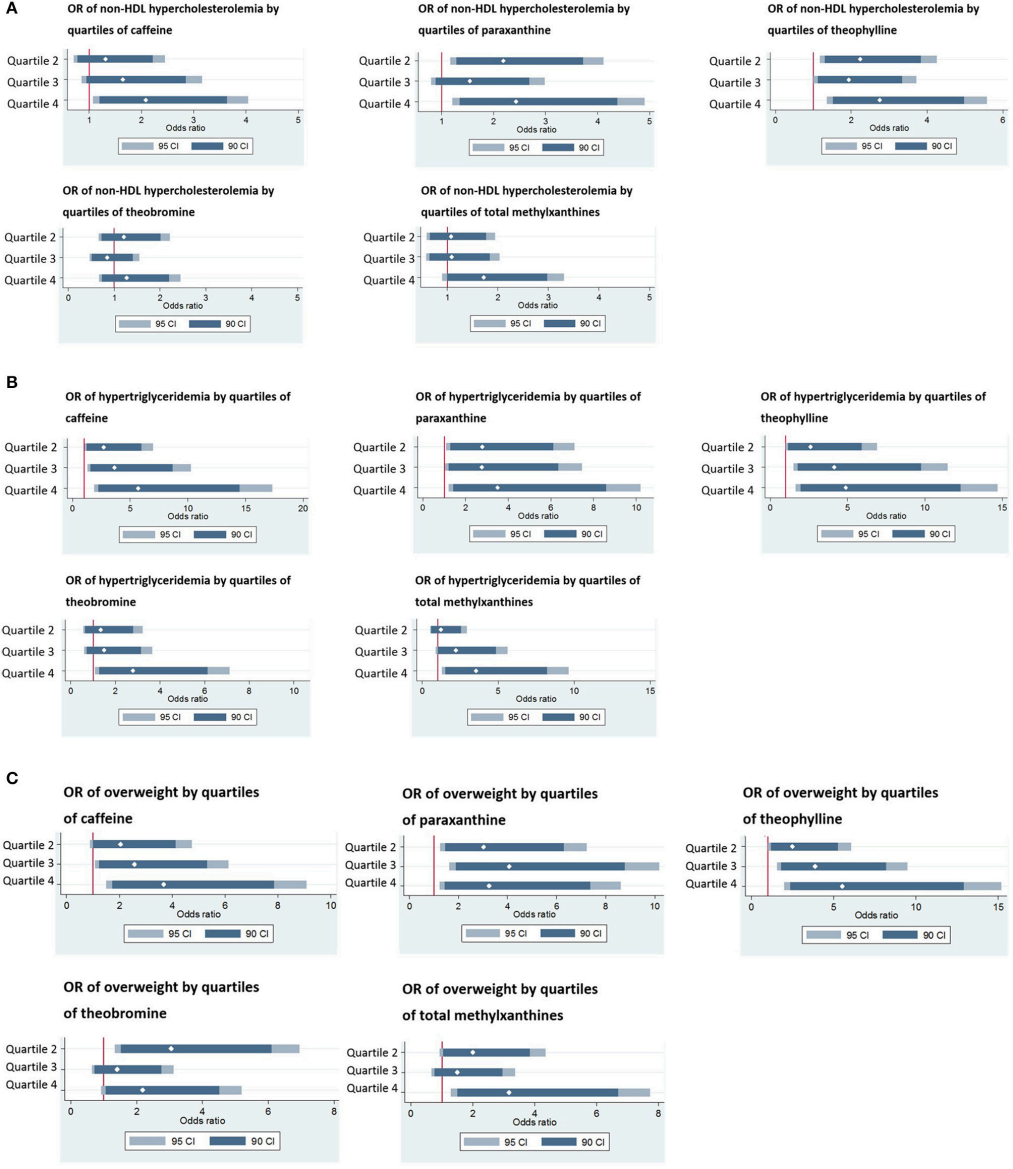
**(A)** Odds ratio of non-HDL hypercholesterolemia (i.e., abnormal levels of plasma non-HDL cholesterol) by quartiles of plasma methylxanthines. Quartile 1 was considered as the reference. Odds ratio were adjusted for age, sex, smoking status, psychotropic medication, treatment duration and BMI. 95 CI: 95% confidence interval. 90 CI: 90% confidence interval. White diamonds represent estimates of adjusted odd ratio. Log caffeine: Q1: ≤ 4.43 ng/ml; Q2: >4.43– ≤ 6.03 ng/ml; Q3: >6.03– ≤ 7.32 ng/ml; Q4: >7.32 ng/ml; Log paraxanthine: Q1: ≤ 5.1 ng/ml; Q2: >5.1– ≤ 6.23 ng/ml; Q3: >6.23– ≤ 7.07 ng/ml; >7.07 ng/ml; Log theophylline: Q1: ≤ 3.43 ng/ml; Q2: >3.43– ≤ 4.31 ng/ml; Q3: >4.31– ≤ 5.16 ng/ml; Q4: >5.16 ng/ml; Log theobromine: Q1: ≤ 5.55 ng/ml; Q2: >5.55– ≤ 6.44 ng/ml; Q3: >6.44– ≤ 7.17 ng/ml; Q4: >7.17 ng/ml; Log total methylxanthine: Q1: ≤ 6.69 ng/ml; Q2: >6.69– ≤ 7.6 ng/ml; Q3: >7.6– ≤ 8.34 ng/ml; Q4: >8.34 ng/ml. Non-HDL hypercholesterolemia was defined as plasma levels of total cholesterol ≥4 mmol/l, according to ESH/ESC guidelines. **(B)** Odds ratio of hypertriglyceridemia by quartiles of plasma methylxanthines. Quartile 1 was considered as the reference. Odds ratio (95% CI) were adjusted for age, sex, smoking status, psychotropic medication, treatment duration, and BMI. 95 CI: 95% confidence interval. 90 CI: 90% confidence interval. White diamonds represent estimates of adjusted odd ratio. Log caffeine: Q1: ≤ 4.43 ng/ml; Q2: >4.43– ≤ 6.03 ng/ml; Q3: >6.03– ≤ 7.32 ng/ml; Q4: >7.32 ng/ml; Log paraxanthine: Q1: ≤ 5.1 ng/ml; Q2: >5.1– ≤ 6.23 ng/ml; Q3: >6.23– ≤ 7.07 ng/ml; >7.07 ng/ml; Log theophylline: Q1: ≤ 3.43 ng/ml; Q2: >3.43– ≤ 4.31 ng/ml; Q3: >4.31– ≤ 5.16 ng/ml; Q4: >5.16 ng/ml; Log theobromine: Q1: ≤ 5.55 ng/ml; Q2: >5.55– ≤ 6.44 ng/ml; Q3: >6.44– ≤ 7.17 ng/ml; Q4: >7.17 ng/ml; Log total methylxanthine: Q1: ≤ 6.69 ng/ml; Q2: >6.69– ≤ 7.6 ng/ml; Q3: >7.6– ≤ 8.34 ng/ml; Q4: >8.34 ng/ml. Hypertriglyceridemia was defined as plasma levels of triglyceride ≥2 mmol/l, according to ESH/ESC guidelines. Only patients in fasting conditions were considered in these analyses. **(C)** Odds ratio of overweight by quartiles of plasma methylxanthines. Quartile 1 was considered as the reference. Odds ratio (95% CI) were adjusted for age, sex, smoking status, psychotropic medication, treatment duration and total cholesterol levels. 95 CI: 95% confidence interval. 90 CI: 90% confidence interval. White diamonds represent estimates of adjusted odd ratio. Log caffeine: Q1: ≤ 4.43 ng/ml; Q2: >4.43– ≤ 6.03 ng/ml; Q3: >6.03– ≤ 7.32 ng/ml; Q4: >7.32 ng/ml; Log paraxanthine: Q1: ≤ 5.1 ng/ml; Q2: >5.1– ≤ 6.23 ng/ml; Q3: >6.23– ≤ 7.07 ng/ml; >7.07 ng/ml; Log theophylline: Q1: ≤ 3.43 ng/ml; Q2: >3.43– ≤ 4.31 ng/ml; Q3: >4.31– ≤ 5.16 ng/ml; Q4: >5.16 ng/ml; Log theobromine: Q1: ≤ 5.55 ng/ml; Q2: >5.55– ≤ 6.44 ng/ml; Q3: >6.44– ≤ 7.17 ng/ml; Q4: >7.17 ng/ml; Log total methylxanthine: Q1: ≤ 6.69 ng/ml; Q2: >6.69– ≤ 7.6 ng/ml; Q3: >7.6– ≤ 8.34 ng/ml; Q4: >8.34 ng/ml. Overweight was defined as body mass index ≥25 kg/m^2^.

Multivariate analyses adjusting for previously mentioned covariates indicated significant associations between plasma methylxanthines and metabolic parameters (S8 Table). In particular, each of the four methylxanthines and their sum (i.e., caffeine, paraxanthine, theophylline, theobromine, and total methylxanthines) were positively associated with TC, HDL-C, and BMI (*p*_corrected_ ≤ 0.02, ≤ 0.02, and ≤ 0.02, respectively), but not with LDL-C and TG. In addition, non-HDL-C was positively associated with all methylxanthines except caffeine (*p*_corrected_ ≤ 0.05). More information are in Supplementary Material. Further adjusted analyses considering methylxanthine quartiles indicated similar findings, showing significant differences of metabolic variables across methylxanthine quartiles (Table 3). In particular, BMI, TC, HDL-C, and non-HDL-C) were significantly increased with increasing total methylxanthine levels (*p*_corrected_ ≤ 0.05). As shown in Figures 1A–C, the odds of displaying non-HDL hypercholesterolemia, hypertriglyceridemia, and overweight was more than doubled for patients in the highest quartile of plasma methylxanthines as compared to those in the lowest quartile (*p*_corrected_ ≤ 0.05, p_corrected_ ≤ 0.03, and p_corrected_ ≤ 0.047, respectively). In particular, compared to patients with caffeine concentration in the lowest quartile, those with caffeine concentration in the highest quartile were twice more likely to display non-HDL hypercholesterolemia (*p*_corrected_ = 0.05), five times more likely to display hypertriglyceridemia (*p*_corrected_ = 0.01) and four times more prone to be overweight (*p*_corrected_ = 0.01).

**Table 3:** Adjusted median of plasma lipid levels according to quartiles of methylxanthine levels.

**Lipid variables**	**Quartile 1**	**Quartile 2**	**Quartile 3**	**Quartile 4**	**p${}_{\text{corrected}}^{\text{a}}$**
**LOG CAFFEINE**					
Total cholesterol, median (IQR) (mmol/l)	4.8 (4.6–5.1)	4.9 (4.6–5.2)	5.1 (4.8–5.4)	5.2 (5–5.5)	**0.02**
LDL cholesterol, median (IQR) (mmol/l)	2.9 (2.7–3.1)	2.9 (2.7–3.2)	3 (2.8–3.3)	3.1 (2.9–3.3)	0.71
HDL cholesterol, median (IQR) (mmol/l)	1.3 (1.2–1.5)	1.3 (1.2–1.5)	1.4 (1.3–1.5)	1.4 (1.3–1.6)	**0.001**
Fasting triglyceride, median (IQR) (mmol/l)	1.3 (1–1.5)	1.4 (1.2–1.7)	1.5 (1.2–1.7)	1.5 (1.3–1.8)	0.15
NonHDL cholesterol, median (IQR) (mmol/l)	3.5 (3.2–3.8)	3.6 (3.3–3.9)	3.7 (3.4–4)	3.8 (3.5–4.1)	0.11
BMI, median (IQR) (kg/m^2^)	24.6 (24.2–25.1)	25.4 (24.9–25.9)	25.7 (25.2–26.2)	25.9 (25.4–26.3)	**0.003**
**LOG PARAXANTHINE**					
Total cholesterol, median (IQR) (mmol/l)	4.8 (4.6–5.1)	4.9 (4.7–5.2)	5 (4.7–5.3)	5.3 (5.1–5.6)	**0.006**
LDL cholesterol, median (IQR) (mmol/l)	2.9 (2.7–3.1)	3 (2.8–3.2)	2.9 (2.7–3.2)	3.2 (3–3.4)	0.71
HDL cholesterol, median (IQR) (mmol/l)	1.3 (1.2–1.6)	1.3 (1.2–1.5)	1.4 (1.3–1.6)	1.5 (1.3–1.6)	**0.001**
Fasting triglyceride, median (IQR) (mmol/l)	1.3 (1–1.5)	1.5 (1.3–1.8)	1.5 (1.2–1.7)	1.5 (1.3–1.7)	0.47
NonHDL cholesterol, median (IQR) (mmol/l)	3.4 (3.2–3.7)	3.6 (3.4–4)	3.6 (3.3–4)	3.9 (3.6–4.1)	0.11
BMI, median (IQR) (kg/m^2^)	24.7 (24.2–25.2)	25.3 (24.8–25.8)	25.6 (25.1–26.1)	25.9 (25.4–26.4)	**0.004**
**LOG THEOPHYLLINE**					
Total cholesterol, median (IQR) (mmol/l)	4.7 (4.5–5)	5 (4.8–5.3)	5 (4.8–5.3)	5.3 (5.1–5.5)	**0.005**
LDL cholesterol, median (IQR) (mmol/l)	2.8 (2.6–3)	3 (2.8–3.3)	3 (2.7–3.2)	3.1 (2.9–3.4)	0.71
HDL cholesterol, median (IQR) (mmol/l)	1.3 (1.2–1.5)	1.3 (1.2–1.4)	1.4 (1.3–1.6)	1.4 (1.3–1.6)	**0.001**
Fasting triglyceride, median (IQR) (mmol/l)	1.3 (1–1.5)	1.4 (1.2–1.7)	1.5 (1.2–1.7)	1.6 (1.3–1.8)	0.08
NonHDL cholesterol, median (IQR) (mmol/l)	3.4 (3.1–3.7)	3.7 (3.4–4)	3.7 (3.3–4)	3.8 (3.6–4.1)	**0.04**
BMI, median (IQR) (kg/m^2^)	24.6 (24.1–25.1)	25.3 (24.8–25.9)	25.8 (25.2–26.2)	25.9 (25.5–26.4)	<**0.001**
**LOG THEOBROMINE**					
Total cholesterol, median (IQR) (mmol/l)	4.9 (4.7–5.1)	5 (4.7–5.3)	5 (4.7–5.3)	5.1 (4.8–5.4)	**0.005**
LDL cholesterol, median (IQR) (mmol/l)	2.9 (2.7–3.1)	3 (2.8–3.3)	3 (2.8–3.3)	3 (2.8–3.2)	0.71
HDL cholesterol, median (IQR) (mmol/l)	1.3 (1.2–1.5)	1.3 (1.2–1.5)	1.4 (1.3–1.6)	1.4 (1.3–1.6)	**0.003**
Fasting triglyceride, median (IQR) (mmol/l)	1.4 (1.1–1.7)	1.4 (1.2–1.7)	1.4 (1.2–1.6)	1.5 (1.2–1.8)	0.46
NonHDL cholesterol, median (IQR) (mmol/l)	3.5 (3.3–3.8)	3.7 (3.4–4)	3.6 (3.3–3.9)	3.7 (3.4–4)	**0.05**
BMI, median (IQR) (kg/m^2^)	24.8 (24.3–25.3)	25.6 (25.1–26.1)	25.5 (25.1–26.1)	25.5 (25–26)	**0.03**
**LOG TOTAL METHYLXANTHINE**					
Total cholesterol, median (IQR) (mmol/l)	4.8 (4.6–5.1)	5.0 (4.7–5.3)	5 (4.7–5.3)	5.3 (5.1–5.6)	**0.005**
LDL cholesterol, median (IQR) (mmol/l)	2.9 (2.7–3.1)	3 (2.8–3.2)	3 (2.7–3.2)	3.1 (2.9–3.3)	0.71
HDL cholesterol, median (IQR) (mmol/l)	1.3 (1.2–1.5)	1.3 (1.2–1.5)	1.4 (1.3–1.6)	1.4 (1.3–1.6)	<**0.001**
Fasting triglyceride, median (IQR) (mmol/l)	1.3 (1–1.5)	1.4 (1.2–1.7)	1.4 (1.1–1.7)	1.6 (1.3–1.9)	0.08
NonHDL cholesterol, median (IQR) (mmol/l)	3.5 (3.2–3.7)	3.6 (3.3–4)	3.6 (3.3–3.9)	3.9 (3.6–4.1)	**0.05**
BMI, median (IQR) (kg/m^2^)	24.7 (24.1–25.2)	25.5 (25–26.1)	25.5 (25–26)	25.8 (25.3–26.3)	**0.004**

*Patients with available age, sex, smoking status, psychotropic medication, treatment duration, BMI and plasma levels of lipids and of methylxanthines, and who did not receive any lipid lowering treatment were included in the analyses. Linear mixed regression adjusting for age, sex, smoking status, psychotropic drug group, treatment duration and BMI were fitted for 630 patients (n observations = 1,272)*.

*^a^p-values for linear trend are reported. Linearity of association was verified. FDR correction was applied. Values in bold are significant*.

*Log caffeine: Q1: ≤ 4.43 ng/ml; Q2: >4.43– ≤ 6.03 ng/ml; Q3: >6.03– ≤ 7.32 ng/ml; Q4 >7.32 ng/ml; Log paraxanthine: Q1: ≤ 5.1 ng/ml; Q2: >5.1– ≤ 6.23 ng/ml; Q3: >6.23– ≤ 7.07 ng/ml; Q4 >7.07 ng/ml; Log theophylline: Q1: ≤ 3.43 ng/ml; Q2: >3.43– ≤ 4.31 ng/ml; Q3: >4.31– ≤ 5.16 ng/ml; Q4 >5.16 ng/ml; Log theobromine: Q1: ≤ 5.55 ng/ml; Q2: >5.55– ≤ 6.44 ng/ml; Q3: >6.44– ≤ 7.17 ng/ml; Q4 >7.17 ng/ml; Log total methylxanthine: Q1: ≤ 6.69 ng/ml; Q2: >6.69– ≤ 7.6 ng/ml; Q3: >7.6– ≤ 8.34 ng/ml; Q4 >8.34 ng/ml*.

Interaction tests were conducted to determine whether any demographic or clinical variable interacted with plasma methylxanthines on metabolic parameters (i.e., on plasma lipid levels and/or on BMI) in order to recognize which stratified analyses would be beneficial. Analyses on plasma lipid levels reported no significant interaction between gender, smoking status, psychotropic treatment duration or psychotropic treatment category, and methylxanthine concentration on lipids, suggesting that stratification by these four variables would not provide additional information and that the association between methylxanthine levels and metabolic parameters appears to be similar across categories of these four variables (S9 Table). However, age and BMI interacted significantly with methylxanthines on plasma lipid levels and smoking interacted significantly with methylxanthines on BMI (S9 Table; S4 Figure). More information are in Supplementary Material.

## Discussion

In the present sample of psychiatric patients, plasma levels of methylxanthines followed an asymmetric right-handed curve, with a small proportion of patients (approximatively half of patients in the fourth quartile, corresponding to around 1/8 of all patients) having particularly elevated methylxanthine concentrations. This right-skewed distribution is in accordance with an observational study conducted in similar settings showing that 13% of psychiatric patients presented a markedly high coffee consumption (24). In the present study, distribution of methylxanthines from blood samples collected in non-fasting conditions was similar than those from blood sample drawn in fasting conditions (data not shown). This suggests that the fasting status did not influence blood levels of methylxanthines.

In accordance with previous studies, metabolic parameters including lipid levels and BMI worsened significantly during treatment with psychotropic drugs (33, 40, 41). In parallel, plasma levels of methylxanthines increased during psychotropic treatment, in accordance with a study suggesting that caffeine consumption may be related to increased thirst due to anticholinergic effects of certain psychotropic drugs (42). In addition, further analyses in the present sample showed that patients receiving drugs with important sedative and anticholinergic effects [i.e., drugs with high concomitant affinities for AchM and H1 receptors, namely clozapine and olanzapine (39)] tended to have higher levels of methylxanthines except theobromine as compared to patients who received drugs with less potent anticholinergic and sedative effects. These findings support the hypothesis of an increased coffee consumption to overcome sedative effects and/or an increased thirst.

The main finding of the present study is that metabolic parameters including BMI and plasma lipid levels were positively associated with plasma levels of methylxanthines in multivariate analyses adjusting for possible confounding variables. These results are consistent with secondary findings from a recent large population-based study on arterial stiffness, showing increased metabolic parameters (i.e., BMI and plasma lipid levels) with increasing quartiles of methylxanthines (43). In addition, our results are in accordance with the most recent meta-analysis evaluating association between coffee consumption and lipid levels, which recognized that, in general population-based cohorts, drinking coffee was associated on average with significant increase of lipid levels (e.g., TC by 0.21 mmol/l) (19). This influence was possibly attributable to biological effects of caffeine (e.g., increased fat oxidation and an increased lipolysis) (20). Of note, in the present study and in agreement with a previous study in the general population (43), increasing quartiles of methylxanthines were associated with higher levels of beneficial cholesterol (i.e., HDL-C). Because TC, TG, and non-HDL-C levels increased to the same extent with increasing methylxanthine quartiles, it seems unlikely that the cardiovascular advantage provided by increased HDL-C levels would counteract detrimental influence of the three other lipid parameters (i.e., TC, TG, and non-HDL-C). Concerning the present positive association between methylxanthine levels and BMI, recent reviews evaluating the influence of coffee consumption on metabolic outcomes observed conflicting results, i.e., that coffee consumption and caffeine intake are associated with a decreased risk for metabolic syndrome (2, 44). Possible mechanisms to explain this reduced risk include that caffeine decreases body weight by increasing energy expenditure (45), and that this molecule induces changes in the structure of gut microbiota (i.e., a decreased Firmicutes to Bacteroidetes ratio), in favor of an anti-obesity profile (46). On the other hand, a recent mendelian randomization study suggested that high caffeine intake is causally associated with high BMI and contrariwise with a lower risk of obesity (47). Thus, the association between methylxanthine levels, BMI, and obesity is still controversial and results from previous studies emphasize the need of future studies to better understand mechanisms underlying these associations.

Plasma levels of methylxanthines increased with the patients' age and reached a plateau around 50 years of age, underlying the importance of considering age as a confounding variable in multivariate analyses. These findings are consistent with population-based studies showing that caffeine intake is age-dependent (48, 49) and with another study showing that the maximum number of cups of coffee per person per day reaches a plateau between 30 and 59 years old (50). In the present study, theobromine levels remained stable across age, showing similar concentrations between teenagers and adults. As coffee intake in teenager individuals was reported to be lower than in adults (42), elevated theobromine concentrations in young patients may be attributed to consumption of other products than coffee, including chocolate foods and beverages, which provide low caffeine but constitute the major source of dietary theobromine (51). Moreover, theobromine accounts for only 7–8% of caffeine metabolism (52, 53) and in the present study, the calculated theobromine/caffeine ratio was close to 1, further supporting that the present plasma levels of theobromine result from other sources than from caffeine metabolism exclusively.

Several interactions were observed between methylxanthines and some clinical variables on metabolic parameters (i.e., on plasma lipid levels and on BMI). For instance, age and BMI interacted significantly with some methylxanthines on some lipid phenotypes, showing that the association between plasma methylxanthines and plasma lipid levels was exclusively applicable in young and lean patients. These findings are consistent with previous studies showing that young and lean patients have a greater risk to develop metabolic side effects than older and heavier patients and that clinical and/or genetic susceptibilities are more easily revealed in these patients than in other patients (33, 34, 54). In the present study, another relevant interaction was observed: positive associations between plasma methylxanthines and BMI were restricted to non-smoking patients. This finding is consistent with another study evaluating the association between caffeine intake and hypertension that reported an influence of caffeine on hypertension in non-smokers but not in smokers (55). One hypothesis to explain this restrictive association is that by inducing CYP1A2 [i.e., the main enzyme responsible for caffeine metabolism (56)], smoking alters plasma concentrations of methylxanthines and may blind the association between methylxanthines and lipid levels. In addition, smoking has been shown to stimulate fat metabolism (57), which can have an impact on BMI and suppress the contribution of methylxanthines on this metabolic variable.

Interestingly, most of our positive findings involved caffeine as well as other methylxanthines (i.e., paraxanthine and theophylline) but not theobromine. These results are in accordance with recent findings (43) and can be partly attributed to the fact that theobromine is less active than caffeine. Thus, theobromine has two- to three-fold lower affinity than caffeine for adenosine receptors A1 and A2A (58) and penetrates the blood-brain barrier less readily than caffeine (59).

Results of the present study should be considered with the following limitations. First, although this study was longitudinal, no link of causality could be drawn between methylxanthines and metabolic parameters. Mendelian randomization analyses would be a valuable approach to evaluate causality. However, much larger sample sizes (typically ≥ 10,000 participants) are required for such analyses (60). Future studies including a higher number of participants should therefore be conducted in order to better understand these associations. Second, although most important factors were considered in the present study, biological half-life of caffeine varies among individuals and is determined by multiple genetic, physiological, and environmental factors (61) that we could not take into account. In addition, time elapsed between the last consumption of caffeine products and the blood draw was not available, which may have increased the variability of methylxanthine levels. However, methylxanthine levels measured at distinct periods of psychotropic treatment in similar individuals were comparable. In addition, considering the sum of all methylxanthines allowed us to take into account inter-individual differences in caffeine metabolism. It must also be mentioned that the present study has been performed in patients receiving psychotropic treatment known to induce metabolic disturbances and the results are therefore not necessarily valid in non-treated psychiatric patients or in patients treated with drugs with no metabolic effects. Strengths of the present study include its naturalistic setting and its relatively large sample size. In addition, plasma levels of methylxanthine were measured instead of using self-reported caffeine consumption, which enhances accuracy of our results.

In conclusion, the present study showed that plasma levels of methylxanthines are positively associated with lipids and BMI in patients receiving psychotropic treatment known to induce metabolic disturbances. In such patients, caffeine consumption could therefore be an additional environmental risk factor that may further worsen their metabolic condition, especially in young patients who are particularly vulnerable to metabolic side effects. However, further research is needed to better understand how the detrimental effect of caffeine on lipids elevation may be counteracted by its beneficial effect on HDL-C. In addition, studies in patients who receive psychotropic treatment inducing metabolic abnormalities and who warrant specific attention (e.g., young patients with elevated coffee consumption) should be initiated to determine whether dietary advices on gradual diminution of coffee consumption should improve their cardiometabolic health.

## Ethics statement

This study was carried out in accordance with the Declaration of Helsinki, the good epidemiological practice written by the Swiss Society of public health, the Swiss law, and local requirements. The study protocol was approved by the Ethic committee of Vaud (CER-VD) with written informed consent from all subjects.

## Disclosure

CE received honoraria for conferences or teaching CME courses from Astra Zeneca, Forum für Medizinische Fortbildung, Janssen-Cilag, Lundbeck, Mepha, Otsuka, Sandoz, Servier, Vifor-Pharma, and Zeller in the past 3 years, and for writing a review article for the journal Dialogues in clinical neurosciences (Servier) He received an unrestricted educational research grant from Takeda in the past 3 years. AvG received honoraria for a conference or workshop participation from Vifor and Schwabe in the previous 3 years. SC received honoraria for teaching CME courses from Forum für Medizinische Fortbildung in the past 3 years.

## Author contributions

CE had full access to all of the data in the study and takes responsibility for the integrity of the data and the accuracy of the data analysis. Study concept and design was provided by CE. Acquisition of data was provided by AG, AL, CD, and by AD, FV, NA, SC, AvG, and PC. Analysis and interpretation was provided by AD, IG, MB, and MG-R. Drafting of the manuscript was provided by AD. Critical revision of the manuscript for important intellectual content was provided by all authors. Statistical analysis was provided by AD and MG-R. CE and PC obtained funding for the study. Administrative, technical, or material support was provided by AvG, PC, and CE.

### Conflict of interest statement

The authors declare that the research was conducted in the absence of any commercial or financial relationships that could be construed as a potential conflict of interest.
