# Supplementary material for: Association Between Plasma Caffeine and Other Methylxanthines and Metabolic Parameters in a Psychiatric Population Treated With Psychotropic Drugs Inducing Metabolic Disturbances
Source: Front Psychiatry. 2018 Nov 9;9:573. doi: 10.3389/fpsyt.2018.00573 (PMC6238296; doi:10.3389/fpsyt.2018.00573)
Supplement: Supplementary file 1 [file Data_Sheet_1.docx]

**SUPPLEMENTARY DATA**

**METHODS**

**Study design**

Monitoring for physical health risk factors included prospective assessments of BMI, waist circumference, fasting glucose, lipid profile, blood pressure and tobacco smoking as described previously ^1^. When a treatment was stopped for more than 2 weeks or if a drug was replaced by another drug listed in **S1 Table**, the follow-up was restarted from baseline. In case of the introduction of a second studied drug, the follow-up was restarted and the last introduced drug considered as the main treatment.

**Quantification of lipids and of methylxanthines in plasma**

Clinical chemistry assays from plasma samples collected before and after January 2009 were performed at the Unit of Pharmacogenetics and Clinical Psychopharmacology and at the Clinical Laboratory of the Lausanne University Hospital, respectively (both laboratories are ISO 15189 certified), using enzymatic colorimetric assays (Roche Modular P chemistry analyzer, Roche Diagnostics, Basel, Switzerland). Coefficients of variation for these assays were of 1.6%, 2% and 2.8% for TC, TG and HDL-C measurements, respectively. The definition of different categories for elevated plasma lipid levels varies slightly between different guidelines and recommendations ^2-4^. Low HDL-cholesterol level, i.e. HDL hypocholesterolemia was defined as < 1 mmol/l, high LDL-cholesterol level, i.e. LDL hypercholesterolemia was defined as ≥ 3 mmol/l, high triglyceride level, i.e. hypertriglyceridemia was defined as ≥ 2 mmol/l and high total cholesterol level, i.e. hypercholesterolemia was defined as ≥ 5 mmol/l, according to ESH/ESC guidelines ^2^. Plasma levels of caffeine, paraxanthine, theophylline and theobromine were quantified by ultra-high performance liquid chromatography (Waters ACQUITY UPLC system) coupled to a tandem quadrupole mass spectrometer (Waters TQD) with electrospray ionization. Limit of quantification for all analytes was 5 ng/ml. The method was validated according to international guidelines using a stable isotope-labeled internal standard for each analyte (detailed method available on request).

**RESULTS**

Multivariate analyses indicated significant associations between plasma methylxanthines and metabolic parameters (**S7 Table**). Given that methylxanthines considered in multivariate models were log-transformed, reported estimates can be interpreted as follows: when plasma levels of methylxanthines doubles or triples, the expected difference in plasma lipid levels corresponds to [estimate] multiplied by *ln(2)* or *ln(3)*, respectively. In particular, since a five-fold increase in plasma levels of total methylxanthines was observed between first and third methylxanthine quartiles, this corresponds to increases of 0.09 mmol/l, 0.04 mmol/l, 0.06 mmol/l and 0.31 kg/m^2^ of TC, HDL-C, nonHDL-C and BMI, respectively.

Interaction analyses showed that age and BMI interacted significantly with methylxanthines on plasma lipid levels and smoking interacted significantly with methylxanthines on BMI (**S8 Table; S4 Figures**). Thus, as illustrated in **S4.1** **Figure**, the association between quartiles of certain methylxanthines and plasma lipids were restricted to patients younger than the median age (i.e. 35 years old). In addition, associations between some methylxanthines and lipid levels were modified by BMI. For instance, influence of theophylline and caffeine quartiles on TC and on HDL-C levels, respectively, were restricted to patients whose BMI was lower than the median BMI (i.e. 24.2 kg/m^2^). (**S4.2** **Figure**). As for BMI outcome, significant interactions between methylxanthine and smoking status on BMI were recognized. Thus, non-smoker patients had gradual increases of BMI with increasing methylxanthine quartiles, whereas patients who smoked were less concerned with the possible influence of methylxanthines on BMI (**S4.3** **Figure**).

Of note, interaction analyses including diagnosis as a covariable showed no interaction between methylxanthines and diagnosis on metabolic outcomes (**S8 Table**), suggesting that the influence of caffeine on metabolic outcomes could be similar across the different psychiatric diseases.

**S1 Table. Drugs included in the metabolic follow-up recommendation**

| **ANTIPSYCHOTICS** | | **ANTIDEPRESSANTS** | | **MOOD STABILIZERS** |
| --- | --- | --- | --- | --- |
|  |  |  |  |  |
| **Atypical  (second-generation)** | **Typical  (first-generation)** | **Tricyclic** | **Other** |  |
|  |  |  |  |  |
| Amisulpride | Chlorprothixene | Amitriptyline | Mirtazapine | Carbamazepine |
| Aripiprazole | Flupentixol | Clomipramine |  | Lithium |
| Asenapine | Haloperidol | Doxepine |  | Valproate |
| Clozapine | Levomepromazine | Imipramine |  |  |
| Lurasidone | Pipamperone | Nortriptyline |  |  |
| Olanzapine | Promazine | Opipramol |  |  |
| Paliperidone | Sulpiride | Trimipramine |  |  |
| Quetiapine | Tiapride |  |  |  |
| Risperidone | Zuclopenthixol |  |  |  |
| Sertindole |  |  |  |  |
|  |  |  |  |  |

According to international recommendations, a metabolic follow-up is ongoing since 2007 at the Department of Psychiatry in the Lausanne University Hospital ^1^, where inpatients and outpatients are prospectively monitored when starting a pharmacological treatment known to have a potential risk to induce metabolic disturbances (i.e. drugs listed above).

**1.** Choong E, Solida A, Lechaire C, Conus P, Eap CB. Follow-up of the metabolic syndrome induced by atypical antipsychotics: recommendations and pharmacogenetics perspectives. *Revue medicale suisse.* 2008;4:1994-1999.

**S2 Table. Lipid-lowering drugs considered as characterizing dyslipidemia**

The list was extracted from^2^.
This list only provides lipid-lowering drugs prescribed in the present psychiatric sample.

**2.** Compendium Suisse des Medicaments. Vol 2016. Bern, Switzerland: Institut Suisse des produits thérapeutiques; 2014

**S3 Table. Evolution of metabolic parameters during psychotropic treatment known to induce metabolic disturbances.**

Patients with available age, sex, smoking status, psychotropic medication, treatment duration, BMI and plasma levels of lipids and of methylxanthines and who did not receive any lipid lowering treatment were included in the analyses. If multiple follow-ups per patient were available, only data from the first follow-up was considered.

^1^ p-values were calculated using ranksum tests (for continuous variables) and chi2 tests (for categorical variables) between baseline versus 1 month of treatment. Values in bold are significant.

^2^ p-values were calculated using ranksum tests (for continuous variables) and chi2 tests (for categorical variables) between baseline versus from 2 to 5 months of treatment. Values in bold are significant.

^3^ p-values were calculated using ranksum tests (for continuous variables) and chi2 tests (for categorical variables) between baseline versus ≥ 6 months of treatment. Values in bold are significant.

^4^ Low HDL-C, high LDL-C, high TG and high TC levels were defined by HDL hypocholesterolemia (<1 mmol/l; 39 mg/dL), LDL hypercholesterolemia (≥3 mmol/l; 116 mg/dL), hypertriglyceridemia (≥2 mmol/l; 177 mg/dL) and hypercholesterolemia (≥ 5 mmol/l; 193 mg/dL), respectively, according to European Society of Hypertension and of the European Society of Cardiology (ESH/ESC) guidelines.

**S4 Table. Clinical and demographic parameters according to quartiles of methylxanthines.**

Patients with available age, sex, smoking status, psychotropic medication, BMI and methylxanthines and who did not receive any lipid lowering treatment were included in analyses (n=630). Only the first observation per patient was considered.

^1^: p-values were calculated using Kruskal-Wallis one-way analysis of variance for continuous variables and chi-squared tests for categorical variables in 630 patients. FDR correction was applied. Values in bold are significant.

^2^: Log caffeine: Q1: 4.43≤ ng/ml; Q2: >4.43-≤6.03 ng/ml; Q3: >6.03-≤7.32 ng/ml; >7.32 ng/ml; Log paraxanthine: Q1: 5.1≤ ng/ml; Q2: >5.1-≤6.23 ng/ml; Q3: >6.23-≤7.07 ng/ml; >7.07 ng/ml; Log theophylline: Q1: 3.43≤ ng/ml; Q2: >3.43-≤4.31 ng/ml; Q3: >4.31-≤5.16 ng/ml; >5.16 ng/ml; Log theobromine: Q1: 5.55≤ ng/ml; Q2: >5.55-≤6.44 ng/ml; Q3: >6.44-≤7.17 ng/ml; >7.17 ng/ml; Log total methylxanthine: Q1: 6.69≤ ng/ml; Q2: >6.69-≤7.6 ng/ml; Q3: >7.6-≤8.34 ng/ml; >8.34 ng/ml.

**S5 Table. Prevalence of dyslipidemia according to quartiles of methylxanthines**

Patients with available age, sex, smoking status, psychotropic medication, BMI and methylxanthines and who did not receive any lipid lowering treatment were included in analyses (n=630). Only the first observation per patient was considered.

^1^: p-values were calculated using chi-squared tests in 630 patients. FDR correction was applied. Values in bold are significant.

^2^: Log caffeine: Q1: 4.43≤ ng/ml; Q2: >4.43-≤6.03 ng/ml; Q3: >6.03-≤7.32 ng/ml; Q4: >7.32 ng/ml; Log paraxanthine: Q1: 5.1≤ ng/ml; Q2: >5.1-≤6.23 ng/ml; Q3: >6.23-≤7.07 ng/ml; >7.07 ng/ml; Log theophylline: Q1: 3.43≤ ng/ml; Q2: >3.43-≤4.31 ng/ml; Q3: >4.31-≤5.16 ng/ml; Q4: >5.16 ng/ml; Log theobromine: Q1: 5.55≤ ng/ml; Q2: >5.55-≤6.44 ng/ml; Q3: >6.44-≤7.17 ng/ml; Q4: >7.17 ng/ml; Log total methylxanthine: Q1: 6.69≤ ng/ml; Q2: >6.69-≤7.6 ng/ml; Q3: >7.6-≤8.34 ng/ml; Q4: >8.34 ng/ml.

Low HDL-cholesterol level, i.e. HDL hypocholesterolemia was defined as < 1 mmol/l, high LDL-cholesterol level, i.e. LDL hypercholesterolemia was defined as ≥ 3 mmol/l, high triglyceride level, i.e. hypertriglyceridemia was defined as ≥ 2 mmol/l and high total cholesterol level, i.e. hypercholesterolemia was defined as ≥ 5 mmol/l, according to ESH/ESC guidelines. Overweight and obesity were defined as body mass index ≥25 kg/m^2^ and ≥30 kg/m^2^, respectively.

**S6 Table. Methylxanthine parameters according to diagnosis and psychotropic medication groups.**

Patients with available age, sex, smoking status, psychotropic medication, BMI and methylxanthines and who did not receive any lipid lowering treatment were included in analyses (n=630). Only the first observation per patient was included.

^1^: p-values were calculated using Kruskal-Wallis one-way analysis of variance. FDR correction was applied. Values in bold are significant.

^2^: psychotropic medications were categorized as follow for weight gain: olanzapine, clozapine and valproate being associated with the highest risk of weight gain; mirtazapine, lithium, risperidone and quetiapine conferring an intermediate risk; aripiprazole, amisulpride, asenapine, haloperidol, lurasidone and zuclopenthixol being at lower risk. ^1^

^3^: psychotropic medications were categorized as follow for lipid deterioration: olanzapine, clozapine, mirtazapine and quetiapine being associated with the highest risk of dyslipidemia; others medications (i.e. aripiprazole, amisulpride, asenapine, haloperidol, lurasidone, zuclopenthixol, lithium and valproate) being associated with a lower risk.^2^

**1.** Leucht S, Cipriani A, Spineli L, et al. Comparative efficacy and tolerability of 15 antipsychotic drugs in schizophrenia: a multiple-treatments meta-analysis. *Lancet.* 2013;382:951-962.

**2.** Diaz FJ, Perez-Iglesias R, Mata I, et al. Using structural equations to test for a direct effect of some antipsychotics on triglyceride levels in drug-naive first-episode psychosis patients. *Schizophrenia research.* 2011;131:82-89.

**S7 Table. Methylxanthine parameters according to psychotropic drugs groups.**

Observations from patients without missing data were included in analyses.

1: includes drugs with low concomitant affinities for AchM and H1 receptors, i.e. amisulpride, aripiprazole, asepanipe, haloperidol, lithium, lurasidone, mirtazapine, paliperidone, quetiapine, risperidone, valproate and zuclopenthixol.

2: p-values were calculated using Wilcoxon-Mann-Whitney tests. FDR correction was applied.

**S8 Table. Association between plasma methylxanthine levels and lipid levels in psychiatric patients receiving psychotropic drugs inducing metabolic side effects.**

Linear mixed models adjusting for age, sex, smoking status, psychotropic drug group, treatment duration and BMI were fitted. FDR correction was applied. P-values in bold are significant.

**S9 Table. Interaction tests between plasma methylxanthines and clinical variables on plasma lipid levels.**

Interaction terms were included in linear mixed models adjusting for age, sex, smoking status, psychotropic drug group, treatment duration and BMI, whenever applicable.

YES: significant interaction.

No: no interaction.

Interaction tests were considered as significant if p-value ≤0.05.


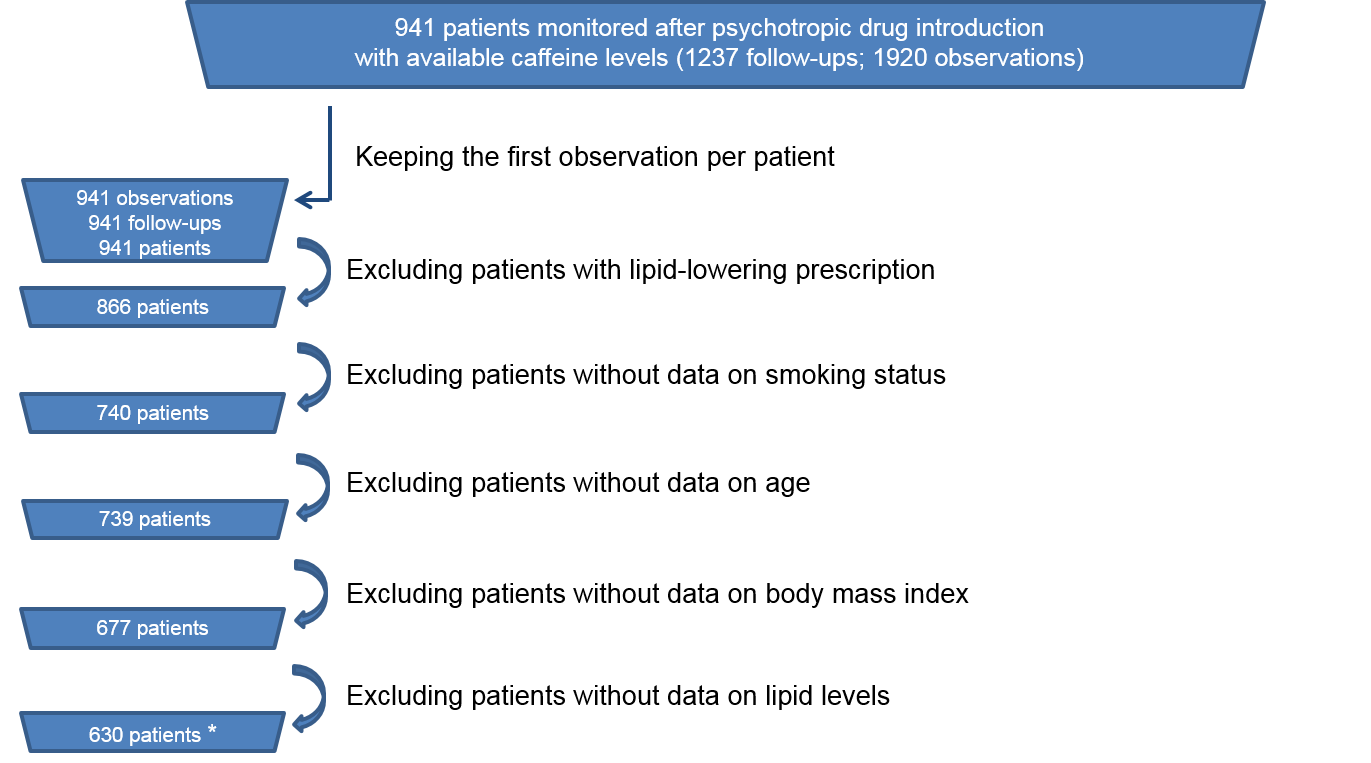


**S1 Figure. Flow chart of patient selection**

* 1274 observations from 630 patients were considered in multivariate analyses. Lipid-lowering drugs are listed in S2 Table


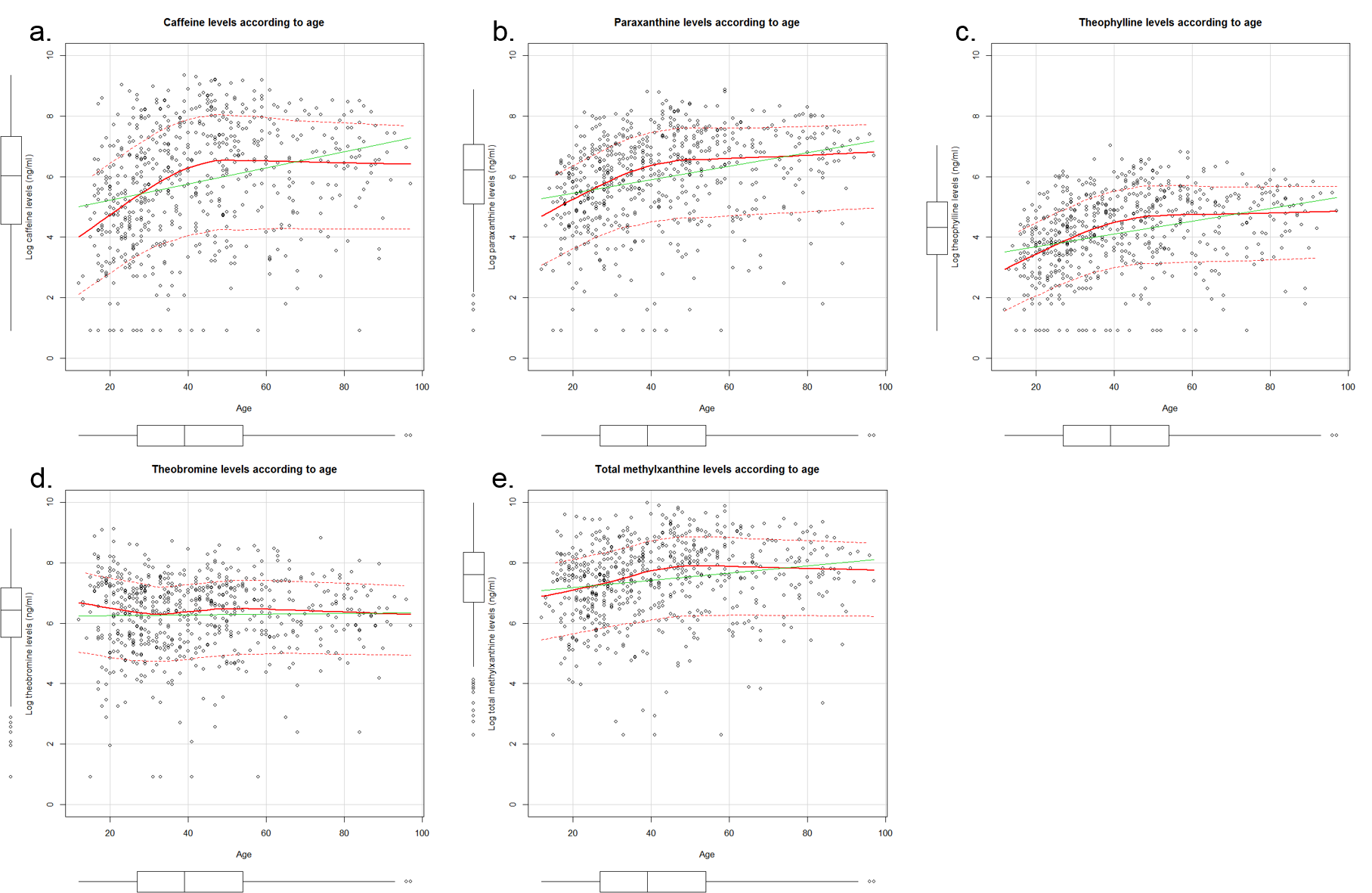


**S2 Figure**. **Methylxanthine levels according to age.**

Regression line is indicated in green; non-parametric regression smooth line is indicated in solid red; smoothed conditional spread is indicated in dashed red. Each point represents a single patient (n=630).

**
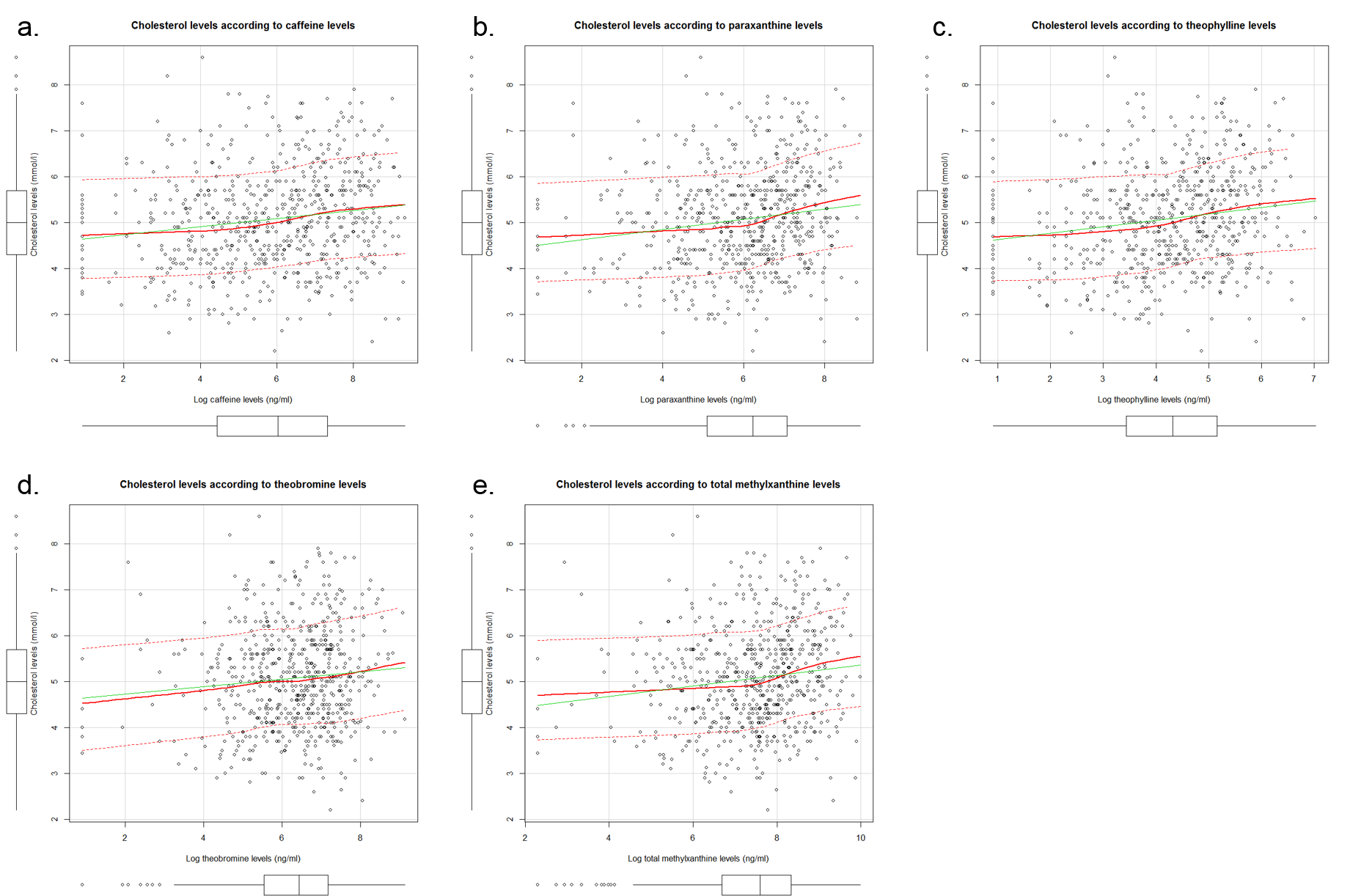
**

**S3.1 Figure**. **Total cholesterol levels according to methylxanthine levels.**

Regression line is indicated in green; non-parametric regression smooth line is indicated in solid red; smoothed conditional spread is indicated in dashed red. Each point represents a single patient (n=630).

**
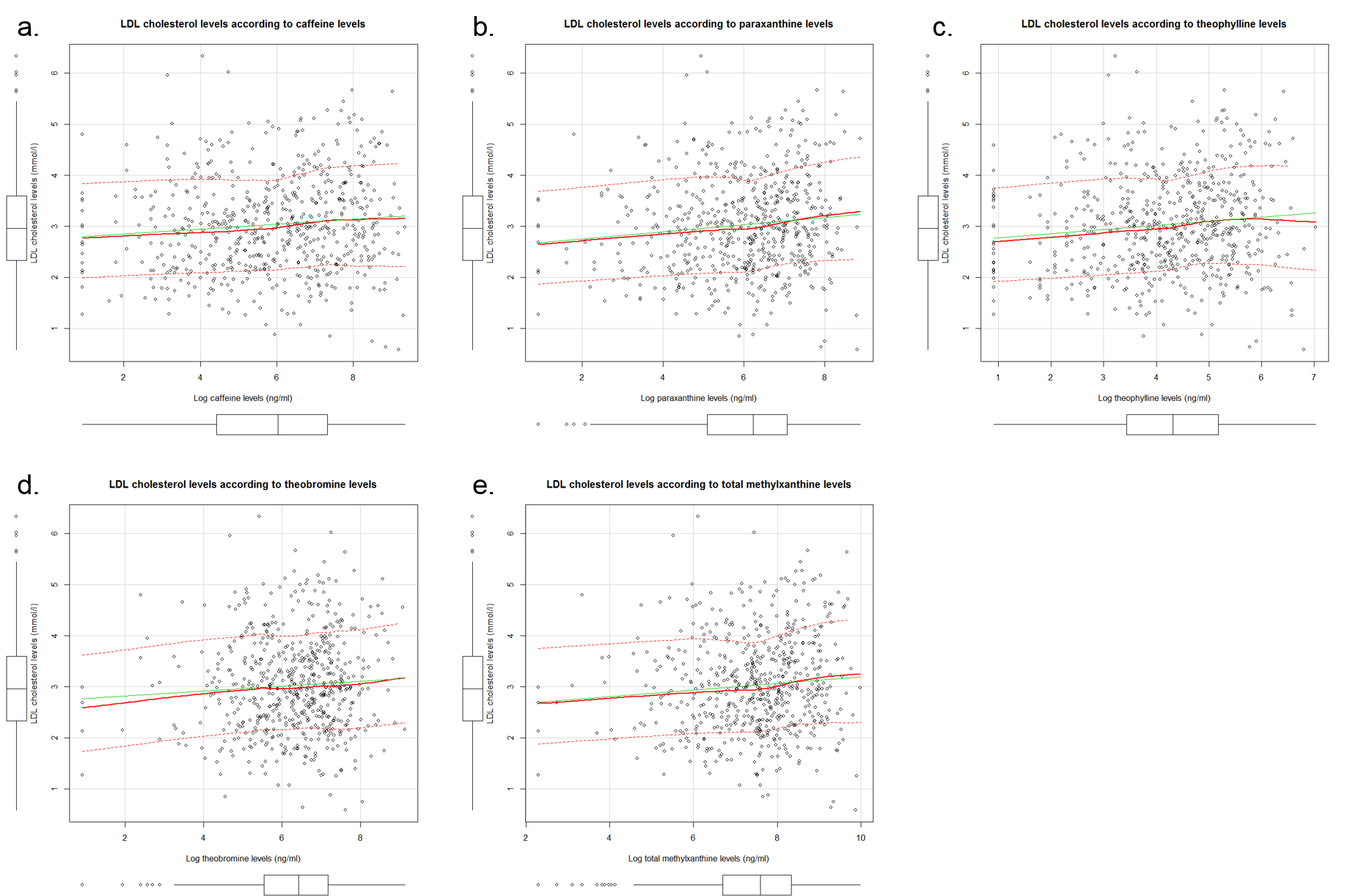
**

**S3.2 Figure**. **LDL-cholesterol levels according to methylxanthine levels.**

Regression line is indicated in green; non-parametric regression smooth line is indicated in solid red; smoothed conditional spread is indicated in dashed red. Each point represents a single patient (n=602).

**
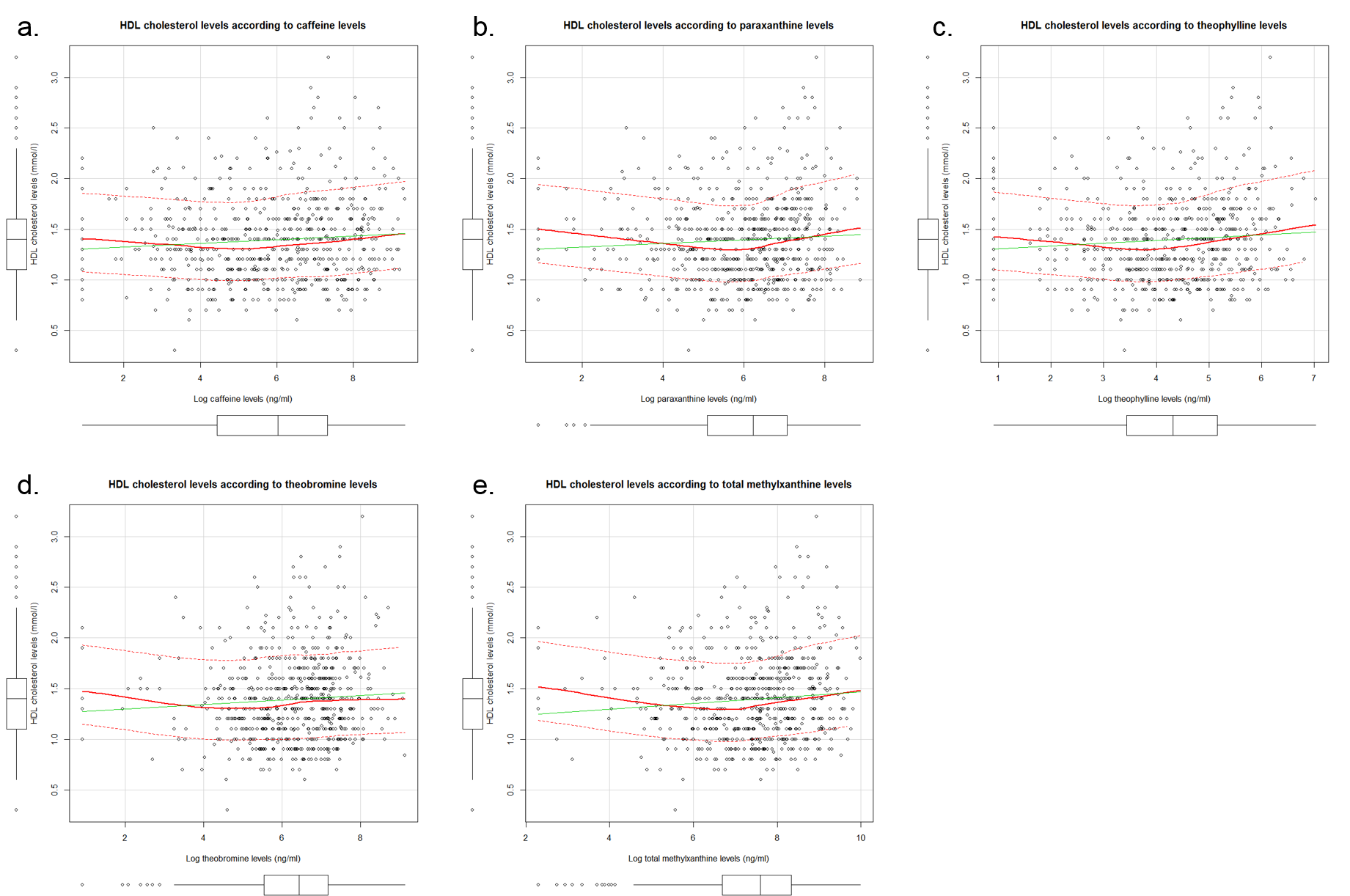
**

**S3.3 Figure**. **HDL-cholesterol levels according to methylxanthine levels.**

Regression line is indicated in green; non-parametric regression smooth line is indicated in solid red; smoothed conditional spread is indicated in dashed red. Each point represents a single patient (n=623).

**
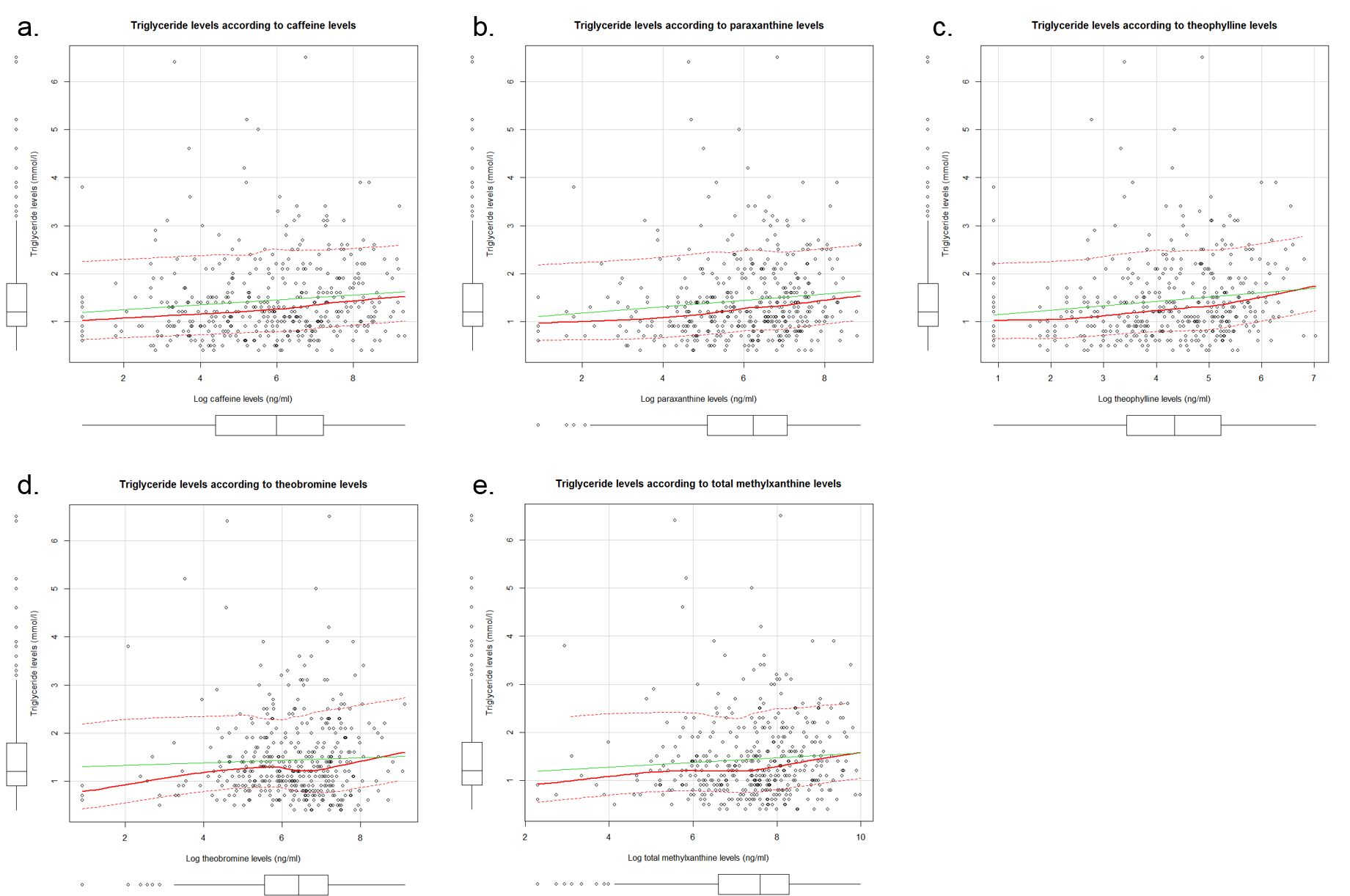
**

**S3.4 Figure**. **Fasting triglyceride levels according to methylxanthine levels.**

Regression line is indicated in green; non-parametric regression smooth line is indicated in solid red; smoothed conditional spread is indicated in dashed red. Each point represents a single patient (n=424).

**
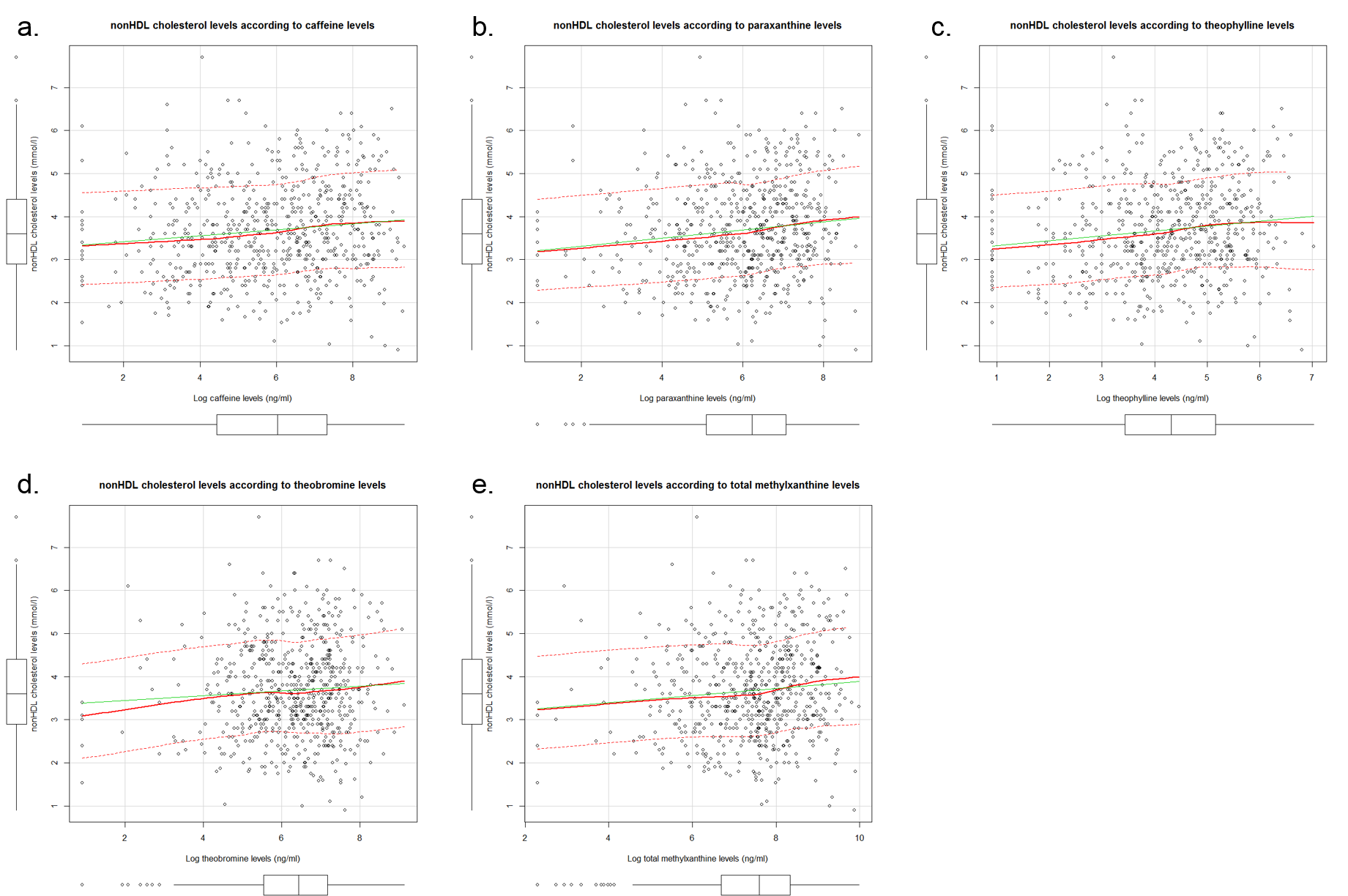
**

**S3.5 Figure**. **NonHDL cholesterol levels according to methylxanthine levels.**

Regression line is indicated in green; non-parametric regression smooth line is indicated in solid red; smoothed conditional spread is indicated in dashed red. Each point represents a single patient (n=623).


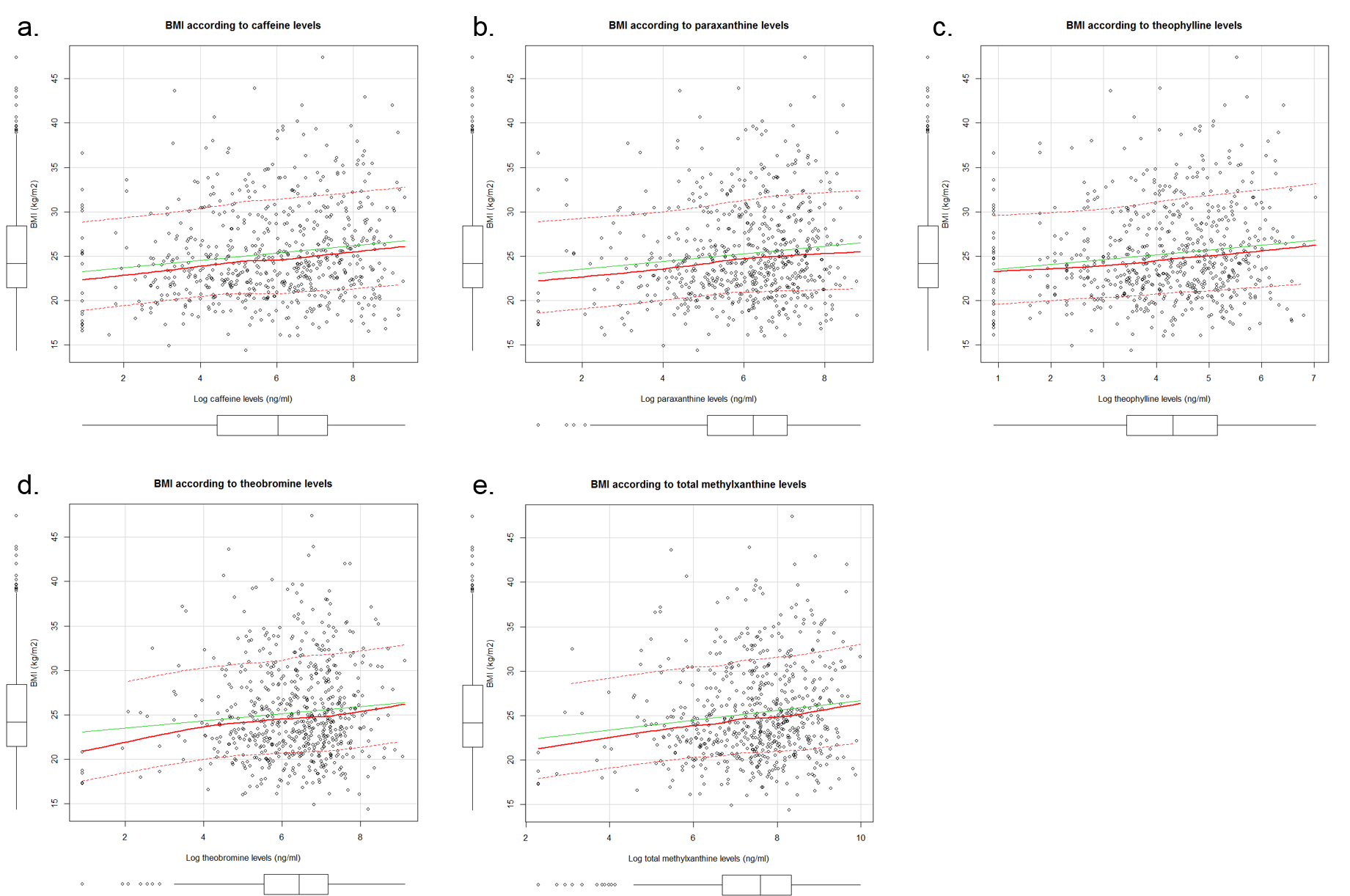


**S3.6 Figure**. **BMI according to methylxanthine levels.**

Regression line is indicated in green; non-parametric regression smooth line is indicated in solid red; smoothed conditional spread is indicated in dashed red. Each point represents a single patient (n=623).

**
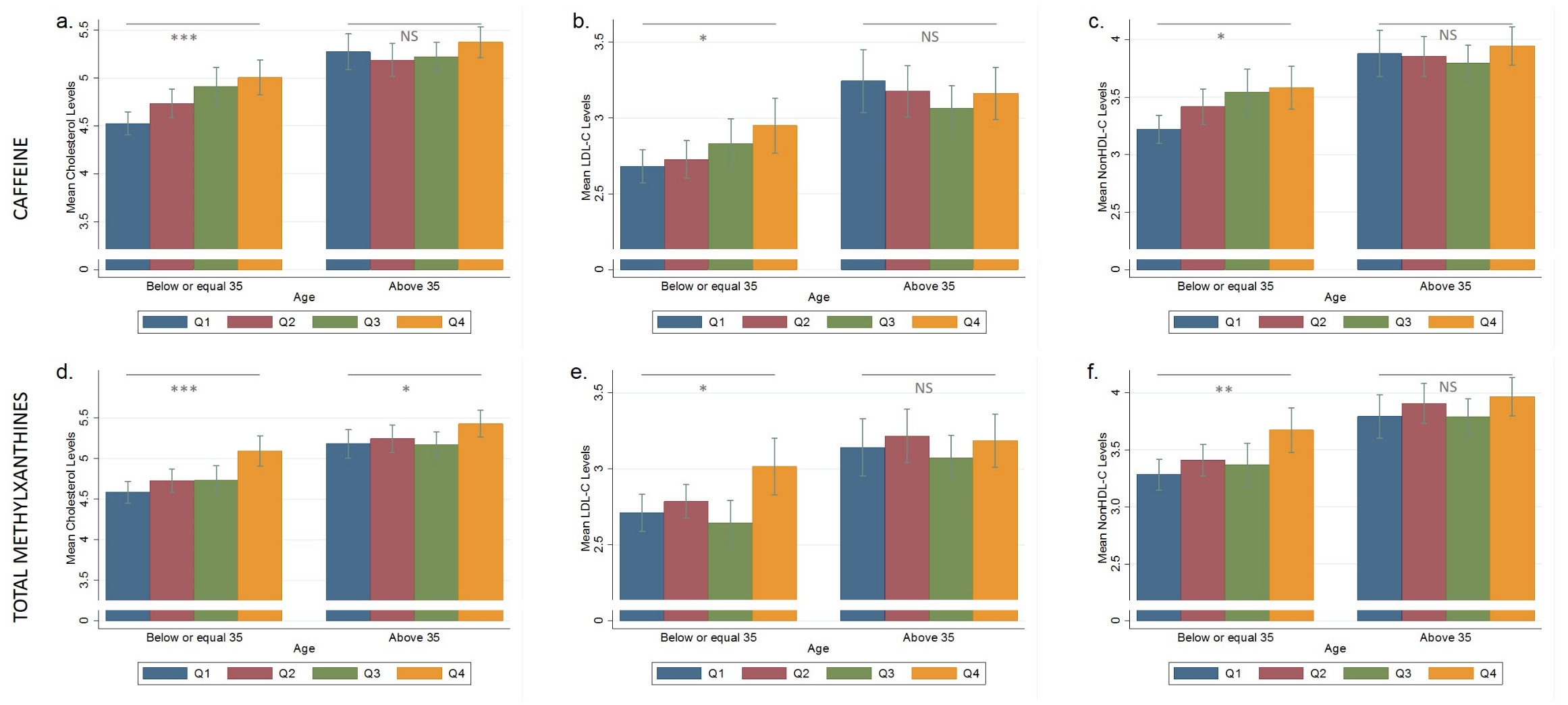
**

**S4.1 Figure**. **Age-stratified distribution of TC, LDL-C and nonHDL-C** **by caffeine and total methylxanthine quartiles.**

a. Age-stratified distribution of TC by quartiles of caffeine. b. Age-stratified distribution of LDL-C by quartiles of caffeine. c. Age-stratified distribution of nonHDL-C by quartiles of caffeine. d. Age-stratified distribution of TC by quartiles of total methylxanthines. e. Age-stratified distribution of LDL-C by quartiles of total methylxanthines. f. Age-stratified distribution of nonHDL-C by quartiles of total methylxanthines.

Log caffeine: Q1: 4.43≤ ng/ml; Q2: >4.43-≤6.03 ng/ml; Q3: >6.03-≤7.32 ng/ml; Q4: >7.32 ng/ml; Log total methylxanthine: Q1: 6.69≤ ng/ml; Q2: >6.69-≤7.6 ng/ml; Q3: >7.6-≤8.34 ng/ml; Q4: >8.34 ng/ml.

Each barplot indicates mean and SD for corresponding plasma lipid levels. Ranksum tests between Q1 and Q4 for corresponding methylxanthines were conducted. FDR correction was applied. NS: non-significant; *: p_corrected_≤0.05; **: p_corrected_≤0.005; ***: p_corrected_≤0.0005.


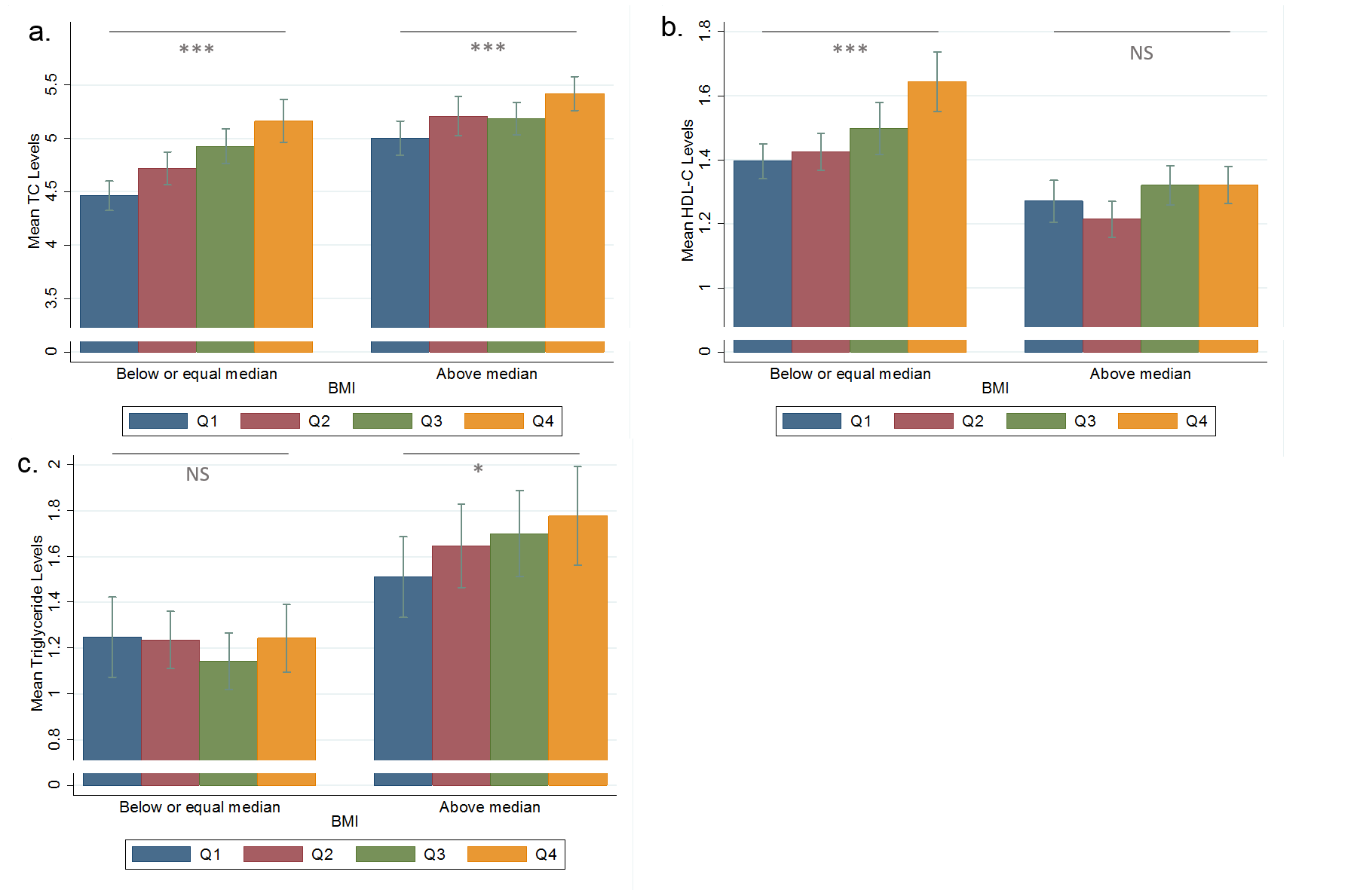


**S4.2 Figure**. **BMI-stratified distribution of lipid parameters by methylxanthine quartiles.**

Median BMI equals to 24.2 kg/m^2^. a. BMI-stratified distribution of TC by theophylline quartiles. b. BMI-stratified distribution of HDL-C by caffeine quartiles. c. BMI-stratified distribution of TG by theobromine quartiles. Log theophylline: Q1: 3.43≤ ng/ml; Q2: >3.43-≤4.31 ng/ml; Q3: >4.31-≤5.16 ng/ml; Q4: >5.16 ng/ml; Log caffeine: Q1: 4.43≤ ng/ml; Q2: >4.43-≤6.03 ng/ml; Q3: >6.03-≤7.32 ng/ml; Q4: >7.32 ng/ml; Log theobromine: Q1: 5.55≤ ng/ml; Q2: >5.55-≤6.44 ng/ml; Q3: >6.44-≤7.17 ng/ml; Q4: >7.17 ng/ml.

Each barplot indicates mean and SD for lipid levels.

Ranksum tests between Q1 and Q4 for corresponding methylxanthines were conducted. FDR correction was applied. NS: non-significant; *: p_corrected_≤0.05; **: p_corrected_≤0.005; ***: p_corrected_≤0.0005.

**
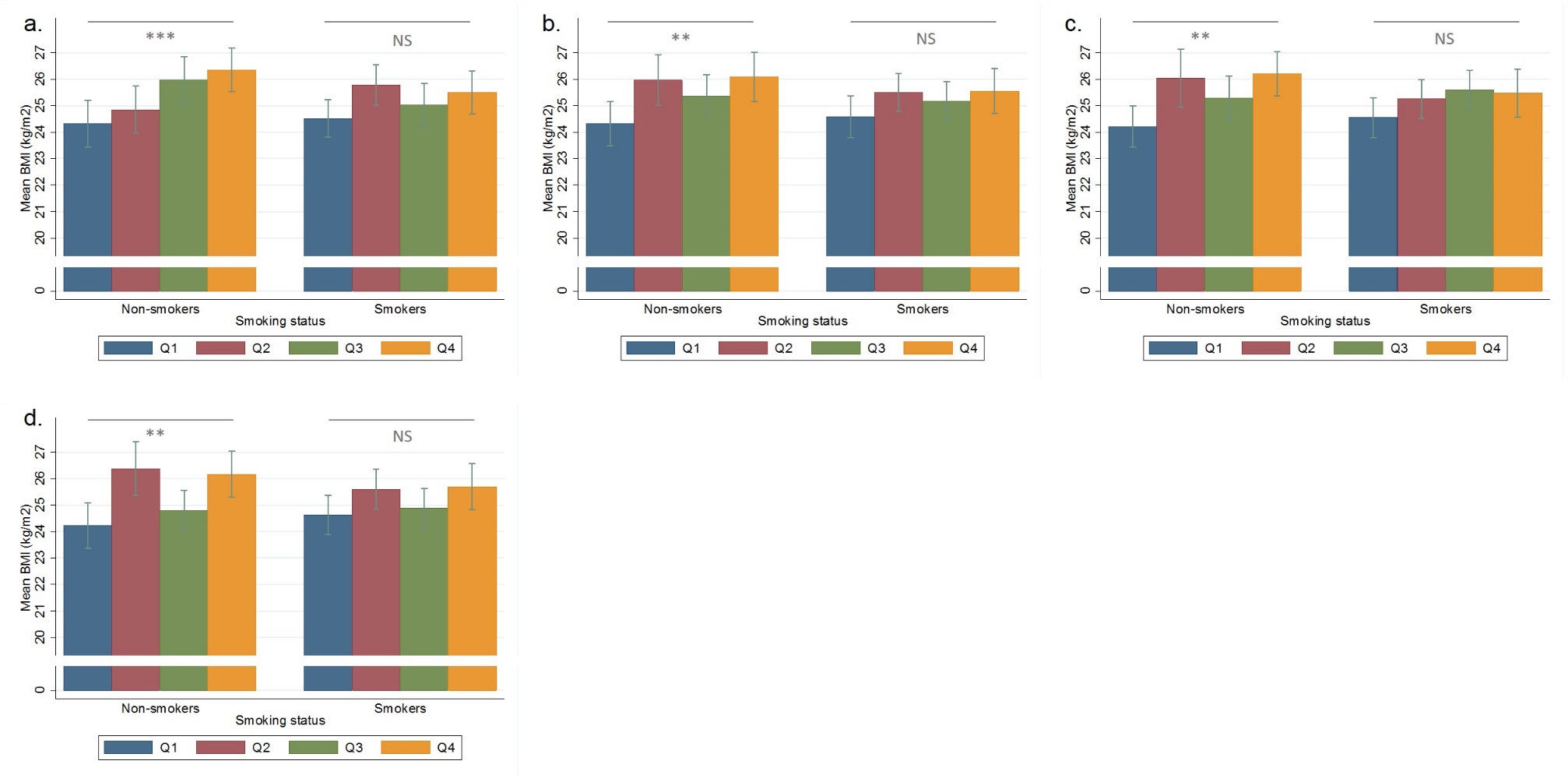
**

**S4.3 Figure**. **Smoking-stratified distribution of BMI by methylxanthine quartiles.**

a. Smoking-stratified distribution of BMI by quartiles of caffeine. b. Smoking-stratified distribution of BMI by quartiles of paraxanthine. c. Smoking-stratified distribution of BMI by quartiles of theophylline. d. Smoking-stratified distribution of BMI by quartiles of total methylxanthines.

Log caffeine: Q1: 4.43≤ ng/ml; Q2: >4.43-≤6.03 ng/ml; Q3: >6.03-≤7.32 ng/ml; Q4: >7.32 ng/ml; Log paraxanthine: Q1: 5.1≤ ng/ml; Q2: >5.1-≤6.23 ng/ml; Q3: >6.23-≤7.07 ng/ml; Q4 >7.07 ng/ml; Log theophylline: Q1: 3.43≤ ng/ml; Q2: >3.43-≤4.31 ng/ml; Q3: >4.31-≤5.16 ng/ml; Q4 >5.16 ng/ml; Log total methylxanthine: Q1: 6.69≤ ng/ml; Q2: >6.69-≤7.6 ng/ml; Q3: >7.6-≤8.34 ng/ml; Q4: >8.34 ng/ml.

Each barplot indicates mean and SD for BMI.

Ranksum tests between Q1 and Q4 for corresponding methylxanthines were conducted. FDR correction was applied. NS: non-significant; *: p_corrected_≤0.05; **: p_corrected_≤0.005; ***: p_corrected_≤0.0005.
